# Nonlinear interaction and compounding factors of vehicle parameters influencing exhaust pollution

**DOI:** 10.1371/journal.pone.0314894

**Published:** 2024-12-18

**Authors:** Augustine Kwame Milku, Charles Atombo, Nana Sarfo Agyeman Derkyi, Francis Attiogbe, Enoch Larson Asuako

**Affiliations:** 1 Regional Center for Energy and Environmental Sustainability, University of Energy and Natural Resources (UENR), Sunyani, Ghana; 2 Department of Mechanical Engineering, Bolgatanga Technical University (BTU), Bolgatanga, Ghana; 3 Department of Civil and Environmental Engineering, University of Energy and Natural Resources (UENR), Sunyani, Ghana; 4 Department of Mechanical Engineering, Ho Technical University (HTU), Ho, Ghana; 5 Department of Renewable Energy Engineering, University of Energy and Natural Resources (UENR), Sunyani, Ghana; 6 Department of Mechanical and Industrial Engineering, University for Development Studies (UDS), Tamale, Ghana; SRM Institute of Science and Technology (Deemed to be University), INDIA

## Abstract

One of the main causes of air pollution, particularly in large cities, is vehicles due to it continued use of hydrocarbon fuels. The understanding of nonlinear interactions of vehicle parameters uncovers more realistic relationships for enhancing formulation of strategies to address vehicle-related pollution. Thus, the study aims to evaluate the interaction and quadratic effect of vehicle parameters on Hydrocarbon (HC), Carbon dioxide (CO_2_), Carbon monoxide (CO), and Nitrogen oxide (NO_x_) emissions. The SV-5Q Vehicle Exhaust Gas Analyzer was used to collect emission concentrations data from one thousand and two (1002) light-duty petrol vehicles at three (3) government-accredited vehicle inspection sites in Accra, Ghana. Pollution control devices, maintenance frequency, and vehicle age were also collected. The linear regression analysis revealed that vehicle age showed a positive linear relationship with CO emissions. Maintenance frequency, on the other hand, demonstrated a negative linear relationship with both CO and HC emissions. The interaction between vehicle age and maintenance frequency positively impacted CO and HC emissions, while the interaction between vehicle age and emission technology had a negative effect on CO. Additionally, the combined effect of frequency of maintenance and emission technology significantly reduced CO emissions but increased HC emissions. Notably, the quadratic effect of vehicle age positively influenced CO emissions. Similarly, CO, HC, and NO_x_ emissions were positively correlated with the squared effect of emission technology. Stricter emissions standards, encouraging frequent maintenance and testing of vehicular exhaust emissions, and doing away with over-aged vehicles are recommended to control and reduce vehicular exhaust emissions.

## 1. Introduction

Exhaust pollution remains one of the environmental and public health concerns, particularly in urban areas where road transport contributes significantly to air pollution [1]. As society is increasingly reliant on cars for daily activities [2], the continued use of hydrocarbon fuels further worsens urban air pollution [3]. The automotive industry has made significant strides in reducing emissions through innovations in engine design, fuel efficiency, and emission control technologies. However, the relationship between vehicle parameters and exhaust emissions is not linear, rather governed by complex, nonlinear interactions. This presents a multifaceted challenge for researchers and policymakers in the road transport sector.

Ghana’s car population has increased significantly in recent years, with the Greater Accra Region housing at least half of the nation’s automobiles [4]. Ghana’s overall population of registered automobiles was estimated to be 2,098,726 in the first quarter of 2017; of the 16 regions in the country, the Greater Accra Region has the most registered vehicles (1,134,599) [5], making up at least 50% of the total number of vehicles in the country [4]. According to the most recent statistics, Ghana has over 2,753,833 million vehicles of various types [6]. As a result, in many developing countries like Ghana, emissions from transportation are a significant source of air pollution making it a source of public concern [7]. Massive emissions of particulate matter made up of black carbon, organic carbon, and other inorganic components, as well as carbon dioxide (CO_2_), carbon monoxide (CO), oxide of nitrogen (NO_x_), unburned hydrocarbon (HC), and other air toxics, are caused by the combustion of fossil fuels from mobile sources [3, 8].

Data from Ghana’s Driver and Vehicle Licensing Authority (DVLA) indicates that there were 84556 motor vehicles registered in total in the city of Accra in 2019 alone. These vehicles come in a variety of sizes, from articulated trucks weighing over 32 tons to cars with engine cubic capacities of up to 2000. Motorcycles, tricycles, tractors, mining equipment, and agricultural implements are not included in this number. Comparably, over 80% of used cars are imported by most African nations, such as Ghana, Nigeria, Ethiopia, and Kenya [9]. Most cars that are sent to Ghana from the Netherlands are between twelve (12) and thirteen (13) years old [10]. This, according to the Ghana Standard Authority (GSA) standard on Road Vehicles—Requirements for imported used motor vehicles are over-aged vehicles as it defines over-aged vehicles as motor vehicles over 10 years, from the date of manufacture [11]. Although cars’ quick speeds make people’s daily lives easier, the vast rise in the number of automobiles on the road has significantly contributed to the pollution of the environment [2]. Travelers are exposed to higher ambient concentrations and exposure rates as a result of the increasing vehicle exhaust emissions [12].

According to a global statistic, over 700 million vehicles are in use globally, and about 50 million motor vehicles are created annually [13]. By 2030, it is expected that there will be about 1300 million cars on the road [14]. According to a study, big cities already have air quality problems as a result of the population’s rapid growth in automobiles [15]. From 2009 to 2015, vehicle exhaust in the US typically accounted for over 60% of NO_x_ and about 26% of VOC. Consequently, the biggest anthropogenic source of CO and other pollutants in the US is motor vehicles [16]. China, on the other hand, only had about 30% and 20%, respectively [17]. According to a report from [18], transportation contributed around 25% of the CO_2_ emissions released by the European Union (EU) in 2019, with road transportation accounting for 71.7% of these emissions. In an effort to lower the levels of air pollution in Europe caused by cars, the EU has imposed "Euro standards" for road vehicle emissions, which establish emission limits for a range of air pollutants, including NO_x_. For light-duty vehicles (LDVs), these standards are Euro 1 to Euro 6 d, and for heavy-duty vehicles (HDVs), they are Euro I to Euro VI-E [19]. A survey in the United Kingdom (UK) also indicated that transport is the largest sector releasing 24% of the Green House Gas (GHG) emissions in 2020, with motor vehicles accounting for 91% of these emissions [20]. According to a study conducted in China, motor vehicle exhaust emissions are a substantial cause of air pollution, resulting in huge health and social costs [21]. The study found that China’s methods for controlling air pollution have changed significantly over the past few decades, with tough enforcement replacing tax enforcement [21].

Ghana is well-known locally for being one of Africa’s fastest-developing nations. [22] and this swift economic growth seems to have contributed to the steady rise in air pollution as a result of a rise in the number of cars [23]. Vehicles’ increasing role in air pollution and greenhouse gas emissions is a serious issue that would negatively impact Ghanaian people’s health [23, 24]. Vehicle exhaust, for example, is Ghana’s largest emitter, according to a report by [25], and it is expected to grow at a rate of 2.3 percent a year. The latest 2023 Annual World Air Quality Report, rigorously compiled by IQAir, paints a bleak picture of Ghana’s air quality, placing it at an all-time worst. Accra, the bustling capital, ranks as Africa’s tenth most polluted city, mirroring a growing worry across the continent. Over the years, Ghana’s air quality has deteriorated. In 2022, Ghana ranked 27^th^ among the world’s most polluted countries. However, the most recent survey ranks Ghana 17^th^, indicating a sharp drop in air quality. The average PM_2.5_ levels, a key indication of air pollution, have been continuously increasing. Large amounts of pollutants are released into the environment every year as a result of a significant increase in the number of outdated, inefficient, poorly maintained, and obsolete automobiles [26].

Advances in emission control technology have had a significant impact on these sources of pollution from automobiles [8]. Although advancing technology for cars alone will not guarantee that emissions will stay low for the duration of the vehicle. This is because fuel and vehicle technology measures will be most effective in reducing emissions if vehicles and parts of the vehicle affecting emissions are properly and routinely repaired and serviced. It is well known that the state of vehicle repair has a significant impact on the amount of pollution generated and of fuel consumed [27].

Despite significant fleet size increases, many metropolitan cities with high vehicle turnover or retrofit rates have seen overall vehicle emission reductions as a result of technological and legislative efforts, especially for light-duty gasoline-powered vehicles [28]. Some developing countries, like Ghana, have limited access to economic tools that encourage the procurement of emission control technologies and fleet rotation programs, which makes it more difficult to achieve reductions in emissions from certain cities [8].

A variety of factors including the technological features of the vehicle fleet, the quality of fuels used, the degree of local development and intensity of economic activity, and the volume of vehicular travel, the emissions contributions from motor vehicles can vary greatly between large cities [8]. Vehicle’s age, size, type, and engine health, as well as the state of its pollution control systems, the vehicle’s maintenance history, and the engine’s characteristics, all affect emissions. The emissions of all motor vehicle classes are increased by a vehicle’s age and inadequate maintenance [29].

Ghana’s emission level is a major cause for concern so determining the impact of the major contributing elements to vehicle exhaust emissions is essential for the effective management and control of vehicular exhaust emissions in Ghana. Therefore, this study was aimed at quantifying the interaction effect of key factors on motor vehicle exhaust emissions in Accra, Ghana. The age of the vehicle, frequency of maintenance, and emission technology were taken into account in this study as the primary predictors of vehicle emissions such as HC, CO, NO_x_, and CO_2_.

It is vital to highlight that the vehicle specifications chosen were based on past related research. A study conducted in Brazil indicated that vehicle maintenance is an important aspect in minimizing pollution emissions [30]. This is because fuel consumption and emission levels are known to be significantly impacted by the state of vehicle repairs [31]. This suggests that old and badly maintained vehicles may be the cause of Ghana’s rising vehicle exhaust emissions. Also, a study has shown that vehicle age and survival functions are important aspects to take into account when calculating fleet average emission factors [32]. An additional investigation by [33] found that the emissions factors in low-income countries are much greater than in high-income countries because the median vehicle age in low-income countries is higher than in high-income countries. It is also important to remember that automakers have looked into many ways to lower vehicle exhaust emissions. Among these techniques, the most effective strategy to reduce the toxic gasses that an internal combustion engine releases is to install a catalytic converter to the exhaust system of the car [34–37].

It is significant to note that the effect of vehicle age, catalytic converter, and maintenance frequency on exhaust emissions is interconnected. Older vehicles are more likely to lack advanced emission control technologies and require more frequent maintenance to ensure optimal performance. Furthermore, the impact of vehicle age and catalytic converter on emissions can be mitigated to some extent through regular and proper maintenance practices. This means that a holistic understanding of the effect of these vehicle parameters is key in formulating comprehensive strategies to address vehicle-related pollution. More so, most existing studies on vehicular exhaust emission focus on linear relationships between vehicle parameters and emissions, which oversimplify the dynamics at play. Notably, exploring nonlinear interactions uncovers more realistic relationships where small changes in vehicle parameters (age, maintenance, catalytic converters) might cause disproportionately large or small impacts on vehicular exhaust emissions. On the basis of the above, this study introduces a novel approach by exploring the interaction and quadratic effect of vehicle parameters on motor vehicle exhaust emissions. Examining these factors collectively, the study seeks to identify potential synergistic and quadratic effects that may impact exhaust emissions beyond the individual influence of each vehicle parameter. This comprehensive analysis will provide a deeper understanding of how these factors interact and contribute to air pollution and how possible is it for Ghana and other developing countries to achieve the United Nations Sustainable Development Goals (SDGs) 3 (Good health and well-being) and 11 (Sustainable cities and communities), offering valuable insights for policymakers and stakeholders in developing effective emission reduction strategies.

## 2. Materials and methods

### 2.1 Study area

The study was carried out in Accra, the capital and largest city of Ghana, which is situated on the Atlantic Ocean’s Gulf of Guinea arm. Located at 5° 33′ N, 0° 13′ W, Accra is a prominent metropolis in West Africa that serves as a political and economic hub of Ghana [38]. Ghana is surrounded by Burkina Faso, Togo, and the Ivory Coast to the west, north, and east, respectively, on the coast of the Gulf of Guinea. The population of Accra is 5,455,692 as of the Ghana Statistical Service’s (GSS) 2021 housing and population census, whereas the total population of the nation is 30,832,019 [39]. Although Accra’s recent growth is encouraging for the country’s economy, more people and activities also entail a greater need for transportation to help people go from one area to another [40]. Many residents of cities and towns like Accra experience challenges getting to and from work in the morning and evening. Other than those who can walk from their homes to their places of employment, many others are unable to avoid utilizing cars [41].

Over the past ten years, there has been a rise in the demand for the usage of automobiles in Ghana due to urbanization and the fact that Greater Accra is the hub of the country’s political, economic, and commercial activity. Out of Ghana’s sixteen (16) regions, the Greater Accra Region had the most registered vehicles (1,134,599) during that time period [5]. Thus, the Greater Accra Region is home to around 50% of automobiles in Ghana [4]. This is due to Accra’s increasing per capita income, which raises people’s purchasing power and causes an increase in the number of cars on the road [42]. This would make urban life even more difficult by increasing automobile pollution, traffic congestion, and metropolitan stress [43, 44] which would lead to serious problems with noise and air pollution [45–47].

### 2.2 Study sample

The probability sampling method was employed in the study to gather data. The techniques of simple random sampling and stratified sampling were also used. To get a comprehensive and well-balanced baseline distribution of vehicle exhaust emissions across the city of Accra, three (3) strata were selected. The strata consist of 415 vehicles from Plaspack Auto Vehicle Inspection Center at Trade Fair-Labadi, 212 vehicles from Awompi DVLA Vehicle Inspection Center at Spintex Road, and 375 vehicles sampled at Vehicle and Inspection Technical Services, Dome. These inspection facilities were chosen because they examine at least not less than one hundred (100) vehicles every day intending to issue a roadworthy certificate that does not involve testing for exhaust emissions.

### 2.3 Experimental setup

The concentrations of CO_2_, HC, CO, and NO_x_ were measured from one thousand and two (1002) light-duty gasoline-powered vehicles using the SV-5Q Vehicle Exhaust Gas Analyzer.

This exhaust gas analyzer has a range of 0 to 1000 (ppm) volume for HC, 0 to 10 (%) volume for CO, 0 to 20 (%) volume for CO_2_, and 0 to 5000 (ppm) volume for NO_x_. It provides a real-time readout of the percentage (%) volume of CO and CO_2_ and the parts per million (ppm) of HC and NO_x_ for gas concentrations. It directly measures the thickness of HC, CO, and CO_2_ in vehicle exhaust using a non-dispersive infrared (NDIR) method. In order to calculate the excess or surplus air coefficient, the density of NO and O_2_ is also examined using an electrochemical sensor. A microcomputer analysis, an on-screen display, and an integrated printer are used for all of this. The operating temperature range for the SV-5Q Vehicle Exhaust Gas Analyzer is -5°C to 50°C, with an operational relative humidity of less than 85% and an atmospheric pressure range of 86.0 to 106 Kpa. The SV-5Q was calibrated at the factory before it was deployed.

The vehicle engines have to be driven for a while to make sure they were at the recommended operating temperature set by the manufacturer because the test is based on a hot start. The vehicles were typically at normal operating temperature when they arrived at the testing and inspection facilities since they had been driven there. The engine on the vehicle was initially run at 2500 rpm for 30 seconds before being dropped to idle in preparation for the test.

Vehicles that were at least five (5) years old at the time of manufacture underwent standard exhaust emissions testing. The standard test measured the concentrations of CO, CO_2_, NO_x_, and HC by inserting a sample probe from the SV - 5Q Vehicle Exhaust Gas Analyzer into the vehicle’s exhaust tailpipe at normal idling speed. The outcomes were noted and printed out as soon as a stable figure was obtained. Vehicles were physically inspected for pollution control devices, and the owners were personally questioned about how often they performed maintenance on the vehicles. During the testing procedure, the concentrations of HC, CO, CO_2_, and NO_x_ were monitored and printed at a 2500 rpm idle speed.

The vehicle parameters that were considered were age, emission technology, and maintenance frequency. To facilitate the analysis, the age of the automobiles was divided into three (3) categories based on the data collected. That is, vehicles in the age range of 5 to 8 were considered as new and coded 1, those in the age range of 9 to 13 are considered moderate and coded as 2, and those in the age range of 14 to 18 were considered to be old vehicles and coded as 3. Two (2) categories were created for the frequency of maintenance: regular and irregular and was coded as 1 and 2 respectively. Three (3) categories were created for the emission technology: 1 denote vehicles without catalytic converter, 2 means vehicles with two-way catalytic converter and 3 represent three-way catalytic converter.

## 3. Results

The ensuing section offers a comprehensive overview of the results obtained, providing valuable insights into the study’s outcomes.

### 3.1 Descriptive statistic

Measurements of HC and NO_x_ concentrations were taken in parts per million (ppm) for each exhaust emission, while CO and CO_2_ concentrations were taken in percentage (%) across 1,002 tests. **Table 1** shows that the average concentration of CO is 11.2%, with a standard deviation of 8.90; CO_2_ is 21.4%, with a standard deviation of 70.13; HC has an average concentration of 1870.2 ppm, with a standard deviation of 1272.41; and NO_x_ is 20 ppm, with a standard deviation of 27.3. The average percentage (%) of vehicular exhaust emission generation is greater for CO_2_ than for CO, while the average part per million vehicle exhaust emission generation is higher for HC than for NO_x_. Additionally, **Table 1** shows the mean and standard deviation of the vehicle parameters.

**Table 1:** Descriptive statistics of emission parameters.

Parameters	Descriptive statistics		Skewness		Kurtosis	
	Mean	SD	Skewness	SE	Kurtosis	SE
**Emission parameters**						
Carbon monoxide (CO %)	11.2	8.9	0.733	0.0773	-0.349	0.154
Carbon dioxide (CO_2_%)	21.4	70.13	21.698	0.0773	481.158	0.154
Hydrocarbon (HC ppm)	1870.2	1272.41	0.376	0.0773	-0.836	0.154
Nitrogen oxide (NO_x_ ppm)	20	27.3	4.314	0.0773	19.945	0.154
**Vehicle parameters**						
Vehicle age	12.26	2.050	-0.386	0.0773	0.077	0.154
Maintenance frequency	1.83	0.376	-1.753	0.0773	1.077	0.154
Emission technology	1.96	0.897	0.071	0.0773	-1.754	0.154

**Fig 1:**
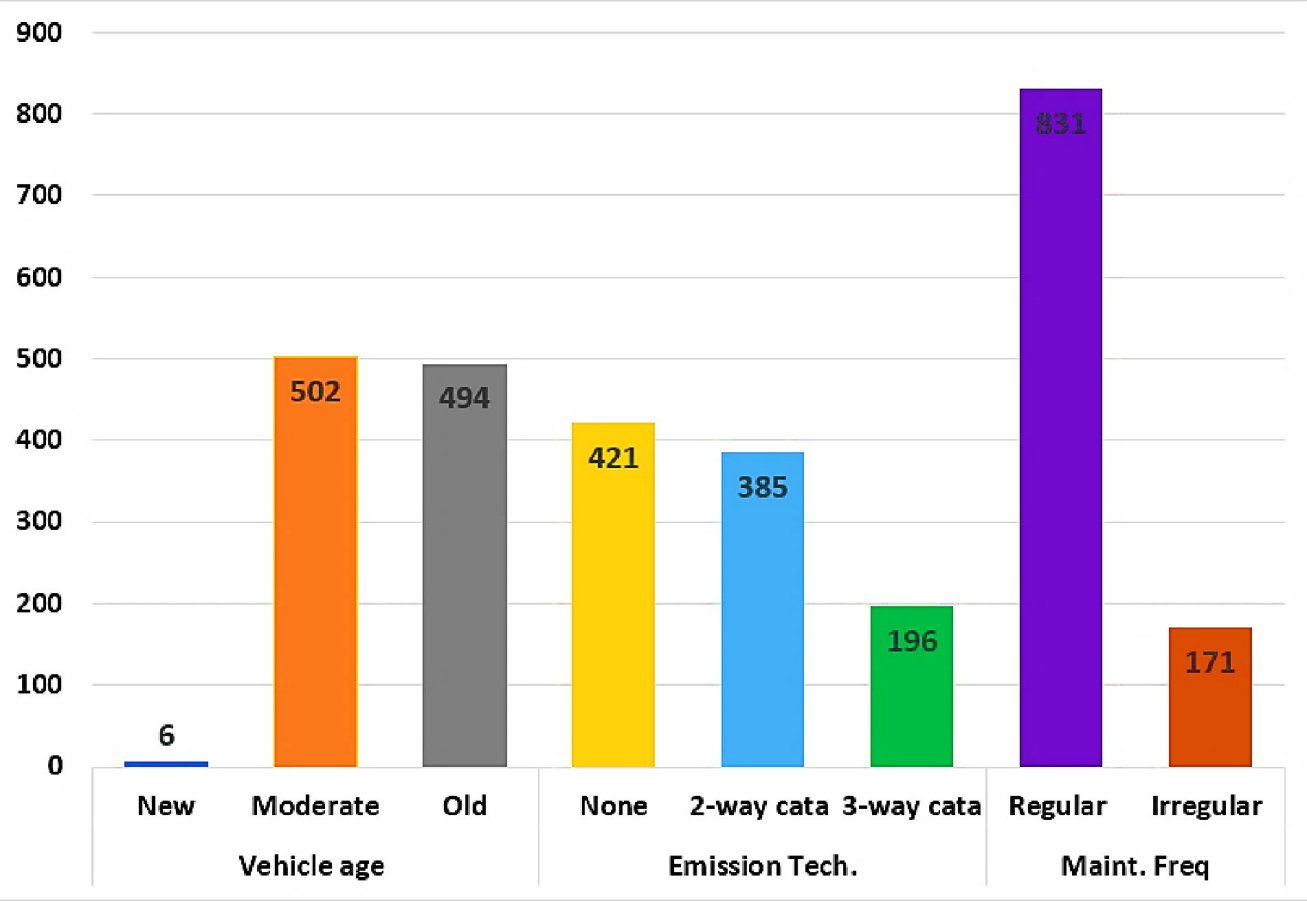
Frequency of vehicle parameters.

As illustrated in Fig 1, the frequency of maintenance sample was divided into two (2) categories, with 171 (17.07%) vehicles recording irregular maintenance and 831 (82.93%) regular maintenance. Furthermore, the sample of vehicle age consisted of three (3) categories: 494 (49.30%) old, 502 (50.10%) moderate, and 6 (0.60%) new automobiles. In addition, the Emission technology sample was divided into three (3) categories, with 385 (38.42%) and 196 (19.56%) vehicles equipped with two-way and three-way catalytic converters, respectively, and 421 (42.02%) vehicles without a catalytic converter.

### 3.2 Linear, interaction, and quadratic effect

The study seeks to assess the interaction and quadratic influence of vehicle parameters on exhaust emissions. Three characteristics were chosen: vehicle age, maintenance frequency, and emission technology, to investigate their interaction and square influence on exhaust emissions of carbon monoxide (CO), carbon dioxide (CO_2_), hydrocarbon (HC), and nitrogen NO_x_. The regression models are evaluated using moderated hierarchical regression analyses, which provide the *P*-value for various response parameters such as CO, CO_2_, HC, and NO_x_ emissions. The unstandardized coefficients with the corresponding *P*-values were reported. The *P*-values less than 0.05 for output responses suggest that the specific vehicle parameter has a significant effect on the output responses. The significant interaction effects are only plotted and further discussed.

To examine the effects of vehicle parameters on exhaust emissions, analyses were performed on each of the exhaust emissions. In each of these regressions, vehicle parameters were entered in the first model. The second model took into account the interaction effect of the age of vehicle on the frequency of maintenance and emission technology, as well as the interaction effect of maintenance on emission technology. The square or quadratic effect of these vehicle parameters was included in the third model to capture the possibility of non-linear relationships between the variables and improve the accuracy of the predictions.

#### 3.2.1 Linear effect of vehicle parameters on exhaust emissions

The result of the linear regression model revealed a strong positive connection between the age of vehicle and the response variable CO (*β* = 3.065; *p*<0.001). The linear effect of maintenance frequency revealed a negative significant connection with CO (*β* = -8.013; *p*<0.01) and HC (*β* = -1903.742; *p*<0.001).

#### 3.2.2 Interaction effect of vehicle parameters on exhaust emission

**Fig 2:**
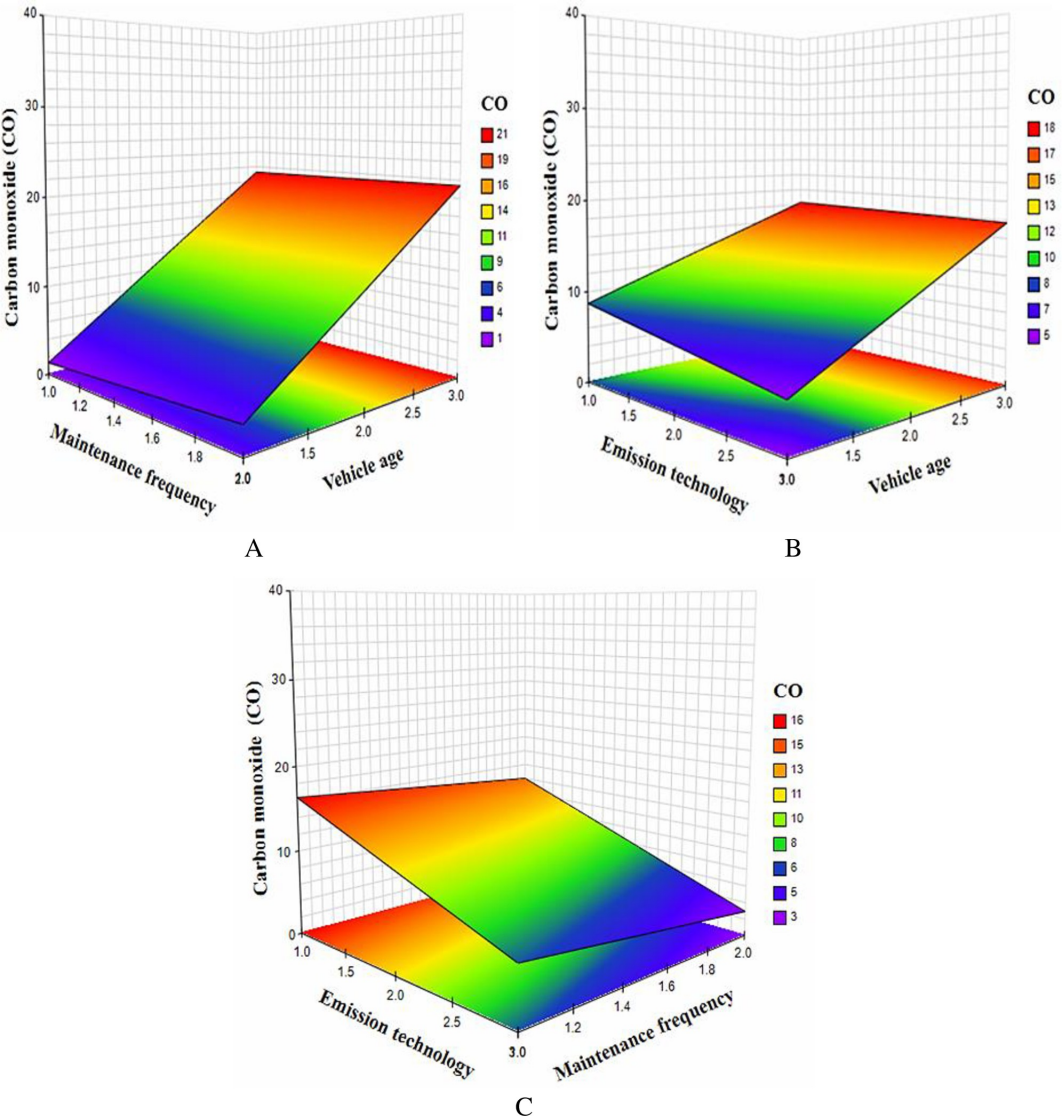
Interaction effect of vehicle parameters on CO. (A) Maintenance frequency by age. (B) Emission technology by age. (C) Emission technology by Maintenance frequency.

After controlling for the main effect, the results of the regression analysis in the second steps showed that the interaction effect of vehicle age and maintenance frequency was positively related to the response variable, CO (*β* = 0.721; *p*<0.001) as shown in **Table 2**. Fig 2A, shows the plot of the combined effect of vehicle age and maintenance frequency confirming that the interaction has a significant relation with CO.

**Table 2:** Moderated hierarchical regression of the effect of vehicle factors on CO and CO_2_.

Vehicle factors	Carbon monoxide (CO)		Carbon dioxide (CO_2_)	
	Coefficient	t-value	Coefficient	t-value
**Linear effect**				
Vehicle Age (A)	3.065***	4.30	-0.857	-0.11
Maintenance frequency (MF)	-8.013**	-3.42	-5.631	-0.22
Emission Technology (ET)	-2.529	-1.04	-4.064	-1.360
**Interaction effects**				
MF x A	0.721***	2.507	-1.205	-0.460
ET x A	0.393*	2.701	0.119	0.110
ET x MF	-1.461*	-2.253	-1.866	-0.290
**Quadratic effect**				
(A)2	0.150***	7.610	0.116	0.550
(ET)2	4.465***	7.380	10.699	1.660
Constant	8.013*	2.420	45.770	1.300

* *p* < .05

** *p* < .01

*** *p* < .001

On the other hand, CO was negatively correlated with the interaction effect of vehicle age and emission technology (*β* = -0.393; *p*<0.05). Besides, the mutual effect of maintenance frequency and emission technology was significant and negatively correlated to CO (*β* = -1.461; *p*<0.05). The interaction plot as shown in Fig 2B and 2C revealed that the interaction effects of emission technology and vehicle age as well as the interaction between emission technology and regular maintenance significantly decrease Carbon monoxide (CO). On the contrary, for vehicles without emission technology and with irregular maintenance, the interaction effect increases the CO. The results further show that the interaction effect of vehicle age and maintenance was positively related to HC (*β* = 58.457; *p*<0.01) as shown in **Table 3**.

**Table 3:** Moderated hierarchical regression of the effect of vehicle factors on HC and NO_x_.

Vehicle factors	Hydrocarbon (HC)		Nitrogen Oxide (NO_x_)	
	Coefficient	t-value	Coefficient	t-value
**Linear effect**				
Vehicle Age (A)	138.666	1.20	0.481	0.160
Maintenance frequency (MF)	-1903.742***	-4.81	-19.113	-1.73
Emission Technology (ET)	-227.863	-0.40	-38.882**	-3.120
**Interaction effects**				
MF **x** A	103.414***	4.52	-0.358	-0.350
ET **x** A	-9.965	-1.06	-0.096	-0.220
ET **x** MF	58.457**	2.51	2.552	1.030
**Quadratic effect**				
(A)^2^	5.969	1.860	0.032	0.400
(ET)^2^	1080.440***	10.960	9.490***	3.810
Constant	5416.280***	10.040	48.380***	3.550

* *p* < .05

** *p* < .01

*** *p* < .001

**Fig 3:**
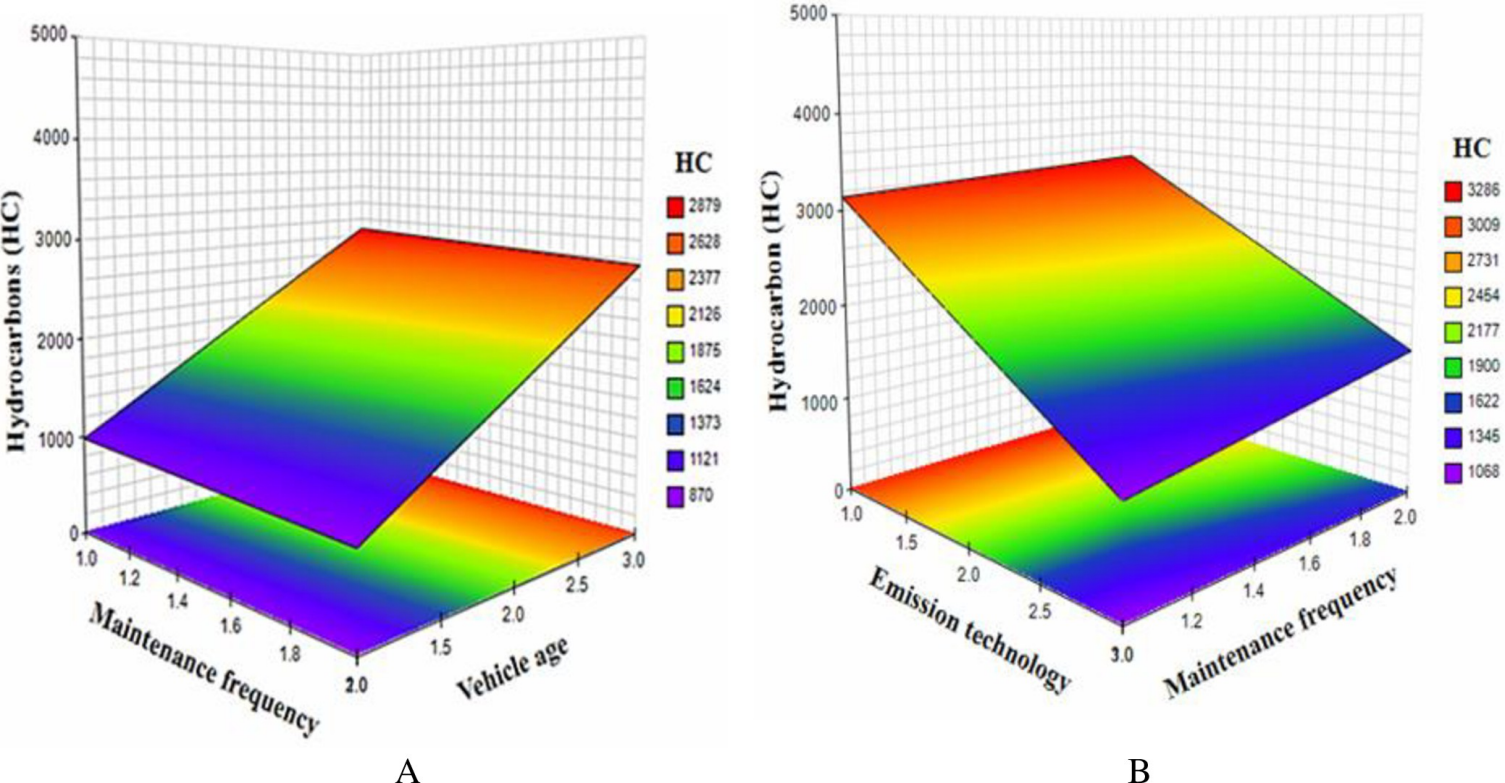
Interaction effect of vehicle parameters on HC. (A) Vehicle maintenance by age. (B) Emission technology by maintenance.

Again, this explains that vehicle age is a significant moderator of the direct relationship between frequency of maintenance and HC. Fig 3A shows the mutual effect of maintenance frequency and vehicle age. It was also observed that the combined effect of maintenance frequency and emission technology was significant and positively related to HC (*β* = 58.457; *p*<0.01).

As shown in Fig 3B, the plot revealed that the mutual effect of emission technology and maintenance frequency has a significant effect on HC.

#### 3.2.3 Quadratic effect

**Fig 4:**
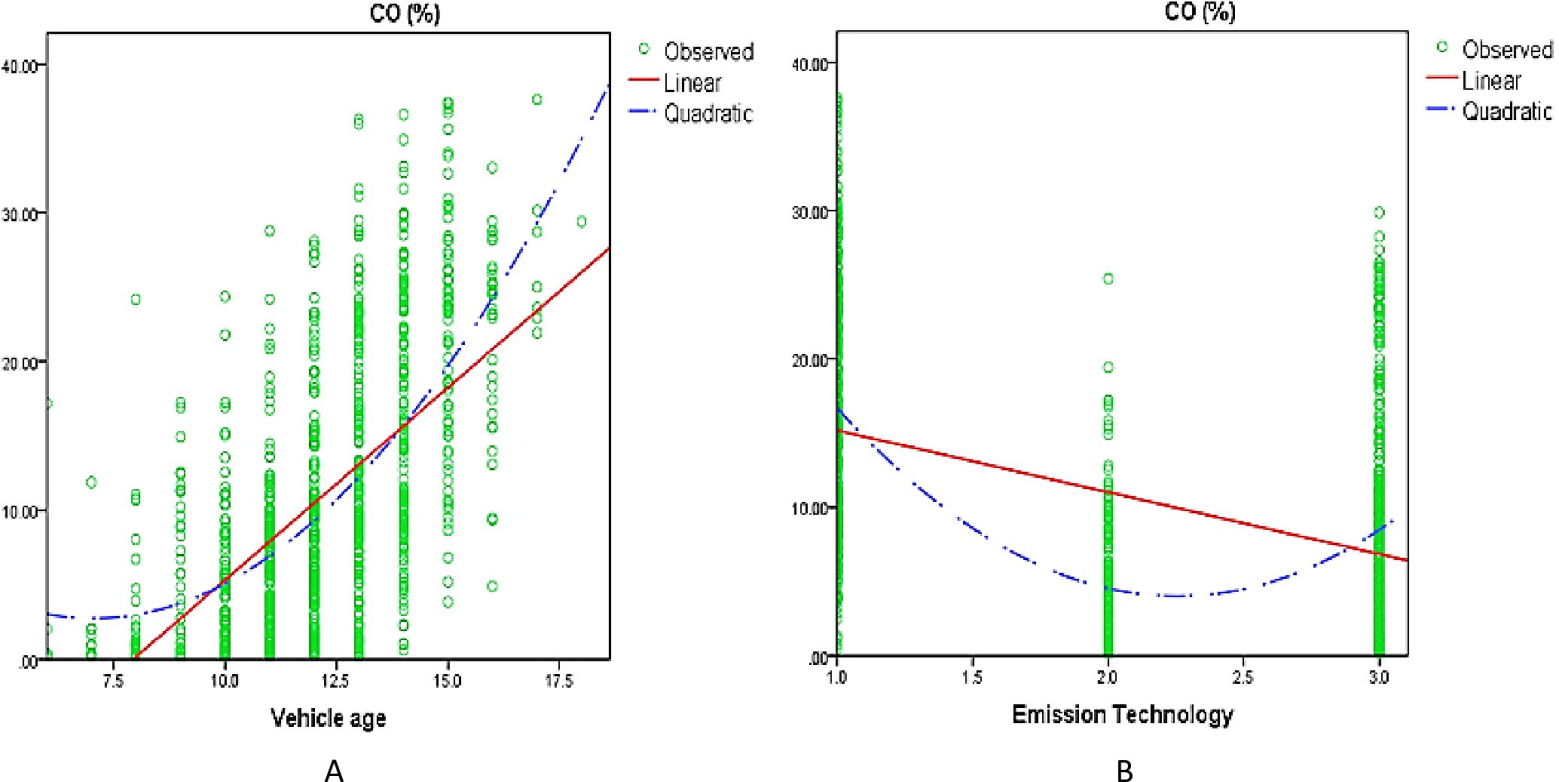
Quadratic plot. (A) Quadratic effect of vehicle age on CO. (B) Quadratic effect of emission technology on CO.

The quadratic analysis was included to capture missing important aspects of the data that were not captured by a linear effect model. Fig 4 shows the quadratic plots of the residuals of CO against vehicle age (Fig 4A) and emission technology (Fig 4B).

**Fig 5:**
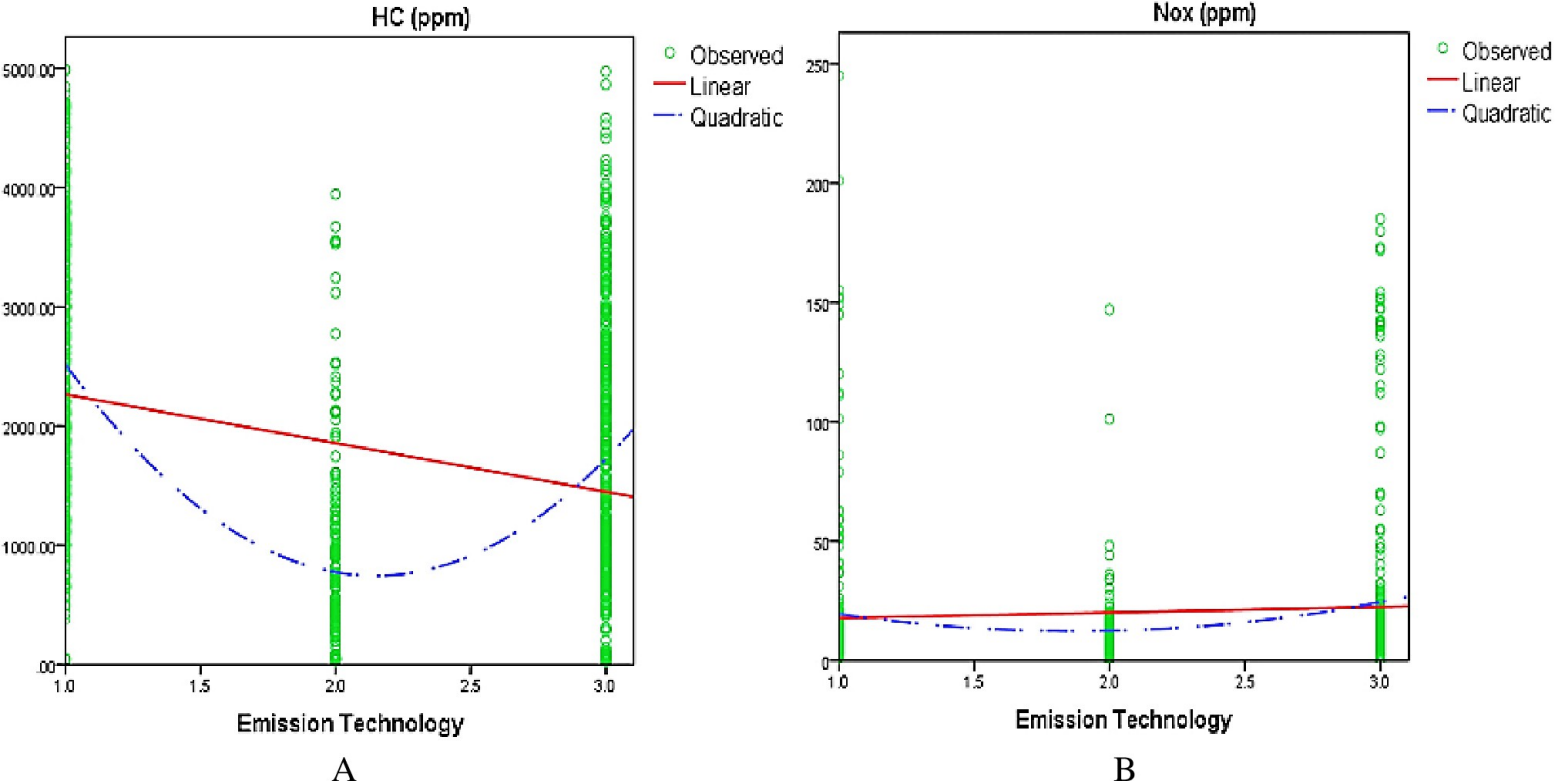
Quadratic plot. (A) Quadratic effect of emission technology on HC. (B) Quadratic effect of emission technology on NO_x_.

Likewise, Fig 5 shows the quadratic plots of the residuals of HC and NO_x_ against emission technology (Fig 5A and 5B). The plots of residuals and fitted values show an upward U-shape pattern, therefore quadratic term for these vehicle parameters (vehicle age and emission technology) was added to the model.

The estimation results for the quadratic effect are reported in **Tables 2 and 3** using two data samples. The estimated results revealed that the quadratic effect of vehicle age had a positive and substantial relationship with the response variable CO emissions. Similarly, the square effect of emission technology was found to be positively correlated with response variables, CO, HC, and NO_x_ emissions.

### 3.3 Model summary

**Table 4** shows that the first and second models had R-squared values of 0.432 and 0.506 for CO and 0.386 and 0.424 for HC. The total p-value (Prob > F) for the model is 0.0000, which is statistically significant at α = 0.05. The R-squared of the third models for CO and HC is 0.665 and 0.593 respectively, which is larger than the first and second models. Given that the CO and HC corresponding *p*-value of the F-statistic is less than 0.05, there is a statistically significant improvement in the third model when compared to the first and second models.

**Table 4:** Model fit summary.

Response variables		Model 1- linear effect	Model 2-interaction effect	Model 3 –quadratic effect
**Carbon monoxide (CO)**	R-squared	0.432	0.506	0.665
	F(df)	208.126(3,998)	134.365(6,995)	118.469(8,993)
	P-value	0.000	0.000	0.000
	R-squared Change		0.074	0.159
	F(df)		29.651(3,995)	72.608(2,993)
	P-value		0.000	0.000
**Carbon dioxide (CO**_**2**_ **)**	R-squared	0.014	0.023	0.029
	F(df)	1.208(3,998)	0.696(6,995)	0.894(8,993)
	P-value	0.306	0.653	0.521
	R-squared Change		0.009	0.006
	F(df)		0.187(3,995)	1.485(2,993)
	P-value		0.905	0.227
**Hydrocarbon (HC)**	R-squared	0.386	0.424	0.593
	F(df)	96.521(3,998)	65.183(6,995)	73.236(8,993)
	P-value	0.000	0.000	0.000
	R-squared Change		0.038	0.173
	F(df)		22.456(3,995)	83.726(2,993)
	P		0.000	0.000
**Nitrogen oxide (NO**_**x**_ **)**	R-squared	0.324	0.337	0.416
	F(df)	6.227(3,998)	2.016(6,995)	5.393(8,993)
	P-value	0.012	0.056	0.001
	R-squared Change		0.013	0.079
	F(df)		0.969(3,995)	9.159(2,993)
	P-value		0.413	0.001

Furthermore, for NO_x_ emission, the R-squared of the first is 0.324, and the model’s *p*-value (Prob > F) is 0.012, indicating significance. The second model has a higher R-squared value (0.337) than the first model, but the *p*-value is insignificant, indicating that there is no significant improvement in the second model over the first model. The *p*-value for the third model shows a significant improvement compared to the first and second models.

However, for CO_2_ models, because the matching *P*-value of the F-statistic for all models was more than 0.05, there is insufficient evidence to imply that the model provides any significant improvement. Concerning CO, HC, and NO_x_ in particular, model 3 provided significant improvement over models 1 and 2, while in the case of CO_2_, the models did not provide a significant improvement.

## 4. Discussions

The linear, interaction, and quadratic effects of motor vehicle parameters on exhaust emissions form the basis of the discussion.

### 4.1 Linear effect of vehicle parameters on exhaust emissions

The result indicates that the age of vehicle corresponds with the increase in Carbon monoxide emission. This result suggests that vehicle age has the potential to contribute to emitting of higher levels of carbon monoxide into the atmosphere. According to a study in Greece, the age of a vehicle has a major impact on its pollution level because the performance of automobiles becomes less effective with age [48]. Similarly, the study of [30] in Brazil also emphasized that vehicle age is the major contributor to vehicular exhaust emissions. In essence, the findings support previous study in China [49] revealing that excessive CO emission generation is due to incomplete combustion associated with the increased age of the engine.

Besides, the frequency of maintenance was negatively related to Carbon monoxide and Hydrocarbons emissions. In this context, maintenance frequency refers to how often vehicles undergo routine maintenance and servicing. This negative relationship suggests that CO and HC emissions drop in proportion to an increase in maintenance frequency. The result confirms the previous assertion that vehicle maintenance is a critical factor for reducing pollutant emissions [30]. In other words, in line with previous findings, such as [50] study conducted in Limpopo Province, South Africa, the present research similarly confirms that vehicles receiving more regular maintenance tend to emit lower levels of CO and HC emissions. This accentuates the importance of proactive maintenance practices in reducing the environmental impact of vehicular emissions.

### 4.2 Interaction effect of vehicle parameters on exhaust emissions

The findings demonstrated the effect of the combined age of vehicle and frequency of maintenance on CO emissions. Clearly, CO emission gradually increased from low to high level of vehicle age whereas it decreased with the regular maintenance frequency. Similarly, a study conducted in the Republic of North Macedonia indicated that the combined effect of irregular maintenance and low vehicle age decreases CO emissions [51]. This means that vehicle age is a significant moderator of the direct relationship between maintenance frequency and CO emissions globally.

More so, the result suggests that vehicles with advanced emission technology and low age decrease CO emissions. The primary source of CO is the incomplete combustion of fossil fuels in automobiles [52, 53]. Therefore, the results imply that older vehicles are less fuel and engine-efficient. These older vehicles are likely to have deteriorated parts and may have resulted to incomplete combustion [54, 55] contributing to more CO in the air. In accordance with a previous study in Poland, it was observed that maintenance frequency is a significant moderator of the direct relationship between emission technology and CO [56].

Furthermore, the interaction plot highlights that HC increased from low to high of vehicle age whereas it remains constant with the maintenance frequency. This explains that vehicles subjected to more frequent maintenance and equipped with advanced emission technologies tend to produce lower CO emissions. In line with a related study in the United States of America, the phenomenon of HC is dominant as the vehicle reaches over age period [57]. This could also be explained that irrespective of the maintenance frequency, vehicle age plays a significant role in regulating HC emissions. In addition, congruent with the study of [58] in some selected cities across the globe, the combination of vehicles without emission technology and with irregular maintenance increases the hydrocarbon (HC) emission of vehicles.

### 4.3 Quadratic effect of vehicle parameters on exhaust emissions

The square effect age of vehicle on CO implies that the relationship between these variables is not a simple linear. Instead, as vehicles age, CO emissions increase, but this increase does not occur at a constant rate. This explains that as vehicles age from new to moderately old, CO emissions might be less as confirmed by a study in Brazil and India respectively [59, 60]. However, after reaching a certain point, further increases in vehicle age might lead to a high concentration of CO emissions. This pattern is possible because newer vehicles might benefit from improved engine technology [61] and better maintenance practices, resulting in lower CO emissions. However, as vehicles age and proper maintenance and emission control measures are neglected, wear and tear will occur as corroborated by [62, 63] in Zurich, Switzerland and Hong Kong respectively. This would deteriorate emission control components and effectiveness, leading to a dramatic increase in CO emissions.

For the automotive industry, the results emphasize the importance of developing vehicles with emission control systems that remain effective as vehicles aged. This could involve designing emission control components that have prolonged durability and robustness. The results in contrast with previous related study conducted in South Africa and Uganda [64, 65], further show that the advancement of catalytic converters within a vehicle’s exhaust system tends to increase the effect of CO, HC, and NO_x_ emissions. Even though increasing the number of catalytic converters in a vehicle can potentially improve emissions reduction efficiency [65], the results suggest that increasing the number of catalytic converters in a vehicle can as well adversely affect emissions, but the impact may depend on several factors including, engine turning, and the efficiency of the catalytic converters. Possibly, the addition of extra catalytic converters may require adjustments to the vehicle’s engine control systems to optimize performance and emissions [66]. Engine tuning and control strategies must consider the increased backpressure caused by the additional converters, which could affect engine efficiency and emissions [67, 68]. This would result in the observed curvilinear trend where these harmful emissions increase. This implies that the need to increase the catalytic converters should be done in accordance with appropriate engine tuning and control to ensure the vehicle continues to perform efficiently and meet emissions standards.

The results could also be explained by the fact that emission technology innovations might lead to substantial reductions in emissions only when the engine attained its maximum operating temperature [69] leading to improved efficiency and cleaner combustion processes [64]. Similarly, studies have shown that, as the engine has not attained its maximum operating temperature, the rate of emissions reduction could slow down [64, 70]. This implies that simply increasing the catalytic converters may not guarantee a reduction in emissions in all engine operating temperatures. Therefore, it is important to further understand the mechanisms behind emission trends in different engine operating temperatures, which could help to develop strategies to enhance emission reduction efforts and ensure that the benefits of advanced emission technologies are sustained over time.

## 5. Conclusion and policy implications

This study explored linear, interaction, and quadratic effects of light-duty gasoline vehicles parameters on exhaust emissions. The findings highlight the urgent need for measures to reduce emissions from automobiles, which contribute to air pollution.

The age of vehicle emerged as a critical factor influencing CO emissions. In addition, maintenance frequency emerged as a powerful mitigating factor, showing a negative relationship with both CO and HC emissions. Vehicles with regular maintenance records produced lower emissions, highlighting the importance of proactive maintenance practices in curbing environmental pollution.

The interaction between vehicle age and maintenance frequency further highlighted the dynamics of CO emissions. It was also evident that maintenance frequency moderates the relationship between vehicle age and CO emissions. Combining improved emission technologies with newer automobiles was found to reduce CO emissions, underscoring the importance of technology in minimizing the negative environmental impact.

Additionally, the study revealed a non-linear, quadratic effect of vehicle age on CO emissions. This finding indicates that as vehicles age, CO emissions gradually increase, but this increase is not uniform. This calls for the implementation of adaptive emission testing procedures that account for the non-linear increase in CO emissions with vehicle age, giving older vehicles more regular testing attention, and promoting fleet turnover by offering financial incentives for replacing older vehicles.

For the automotive industry, these findings underscore the importance of developing emission control systems that remain effective as vehicles age. The durability and resilience of emission-control components can significantly reduce emissions from older automobiles. Furthermore, the study highlighted the positive impact of catalytic converters on CO, HC, and NO_x_ emissions. However, the effectiveness of these technologies is contingent on the engine turning and operating temperature. Therefore, appropriate engine tuning is essential when number of catalytic converters has to be added to ensure the vehicle continues to perform efficiently and meet exhaust emission standards. In addition, understanding the mechanisms governing emission level at different operating temperatures is essential for enhancing emission reduction efforts and ensuring the sustained benefits of advanced emission technologies.

In particular, the study’s findings contribute to a better understanding of the intricate link between vehicle parameters and emissions. It emphasizes the need for proactive maintenance practices, the significance of emission technology in lowering emissions, and the challenges associated with aging vehicles.

To lessen the negative environmental effects of aging automobiles, officials should explore tougher emissions standards, as well as promoting frequent vehicle maintenance and emission testing. High-emission automobiles that fail emission tests in their home country should not be imported. Furthermore, allowing only vehicles with improved emission control features into the country can cut vehicular exhaust emissions. More so, doing away with over-aged vehicles and enhancing public awareness efforts are recommended to control and reduce pollutants emanating from the tailpipe of vehicles. If this is implemented as recommended, it will lead to the attainment of the SDGs 3 (Good health and well-being) and 11(Sustainable cities and communities) in Ghana and other developing countries in Africa and beyond that still depend on used imported vehicles that fail emission test in their country of origin.

Although the study has made significant contributions to the available literature, it is not an end in itself. The usage patterns of vehicles (commercial and private vehicles), were not considered. However, these factors significantly impact the representativeness of emission data. Commercial vehicles typically have higher usage rates, leading to increased wear and tear, more frequent maintenance issues, and higher emissions compared to private vehicles. Moreover, the operating conditions and maintenance schedules for these vehicle types also vary significantly. Therefore, future studies should take these factors into consideration. Future studies should integrate environmental data with vehicle parameters, using techniques such as geospatial modelling or environmental regression models to examine how external conditions moderate emissions. This approach would also provide more region-specific policy recommendations.
